# *MetastaSite*: Predicting metastasis to different sites using deep learning with gene expression data

**DOI:** 10.3389/fmolb.2022.913602

**Published:** 2022-07-22

**Authors:** Somayah Albaradei, Abdurhman Albaradei, Asim Alsaedi, Mahmut Uludag, Maha A. Thafar, Takashi Gojobori, Magbubah Essack, Xin Gao

**Affiliations:** ^1^ Computer Electrical and Mathematical Sciences and Engineering Division (CEMSE), Computational Bioscience Research Center (CBRC), King Abdullah University of Science and Technology (KAUST), Thuwal, Saudi Arabia; ^2^ Faculty of Computing and Information Technology, King Abdulaziz University, Jeddah, Saudi Arabia; ^3^ Al-Hada Armed Forces Hospital, Taif, Saudi Arabia; ^4^ King Saud Bin Abdulaziz University for Health Sciences, Jeddah, Saudi Arabia; ^5^ King Abdulaziz Medical City, Jeddah, Saudi Arabia; ^6^ College of Computers and Information Technology, Taif University, Taif, Saudi Arabia

**Keywords:** machine learning, deep learning, artificial intelligence, metastasis, metastasis site, gene expression, clinical decision-making

## Abstract

Deep learning has massive potential in predicting phenotype from different omics profiles. However, deep neural networks are viewed as black boxes, providing predictions without explanation. Therefore, the requirements for these models to become interpretable are increasing, especially in the medical field. Here we propose a computational framework that takes the gene expression profile of any primary cancer sample and predicts whether patients’ samples are primary (localized) or metastasized to the brain, bone, lung, or liver based on deep learning architecture. Specifically, we first constructed an AutoEncoder framework to learn the non-linear relationship between genes, and then DeepLIFT was applied to calculate genes’ importance scores. Next, to mine the top essential genes that can distinguish the primary and metastasized tumors, we iteratively added ten top-ranked genes based upon their importance score to train a DNN model. Then we trained a final multi-class DNN that uses the output from the previous part as an input and predicts whether samples are primary or metastasized to the brain, bone, lung, or liver. The prediction performances ranged from AUC of 0.93–0.82. We further designed the model’s workflow to provide a second functionality beyond metastasis site prediction, i.e., to identify the biological functions that the DL model uses to perform the prediction. To our knowledge, this is the first multi-class DNN model developed for the generic prediction of metastasis to various sites.

## 1 Introduction

Precision medicine is a path that could profoundly change and improve medical practices. This idea proposes using genetic data of individual patients to enhance clinical decision-making, and “omics” technologies now provide a means to acquire such patient data, making precision medicine feasible. Clinical decision-making includes diagnosis, prognosis, choosing the most appropriate treatment, etc. One avenue pursued to support clinical decision-making is building classifiers using gene expression profiles that can function as forms of artificial intelligence (AI).

Many machine learning methods, including support vector machines, random forest, and boosting, are among the primary tools currently being used to make biological discoveries from the vast amount of available gene expression data (Libbrecht and Noble, 2015). However, deep learning (DL) is emerging as a more powerful machine learning method (Goodfellow et al., 2016), although the primary DL application domain is image recognition and speech recognition. Nonetheless, DL is showing promise in many other fields of science, especially in precision medicine and genomics data analysis (Grapov et al., 2018; Martorell-Marugán et al., 2019), as DL can extract intricate structures in high-dimensional data (Najafabadi et al., 2015). However, DL is still new in the bioinformatics community; thus, only a few published works show its application to gene expression-based models (Daoud and Mayo, 2019). Furthermore, unlike images or text data, gene expression data has no clear structure that we can exploit in a neural network architecture. Thus, many new architectures are surfacing for metastasis prediction from gene expression data, such as multilayer perceptron architecture (Albaradei et al., 2019; Albaradei et al., 2021a; Albaradei et al., 2021b) , autoencoder architectures (Sharifi-Noghabi et al., et al.; Albaradei et al., 2021c; Fakoor et al., 2013) and Graph deep learning (Xu et al., 2021). Most of these proposed models try to solve a binary classification problem that classifies samples as metastatic or non-metastatic (Albaradei et al., 2021a). However no generic computational framework based on DL that accepts raw gene expression data to predict whether cancer is primary or has spread to various metastasis sites exists.

The main concern of DL used in medical applications is the lack of interpretability. The reason being, DL networks can be viewed as black boxes that form an input layer (wherein we place the gene expression profile of patients) and an output layer (offering predictions without interpretability). Suppose we do not meet this interpretability criterion at a good standard. In that case, physicians will not be able to trust the decision of the neural network, as they need interpretable data to ensure patients’ safety. Specifically, they need data about neurons, genes, and related biological processes involved in the prediction and the decision-making process to make informed decisions. Thus, researchers are now attempting to make the DL networks more interpretable.

**FIGURE 1:**
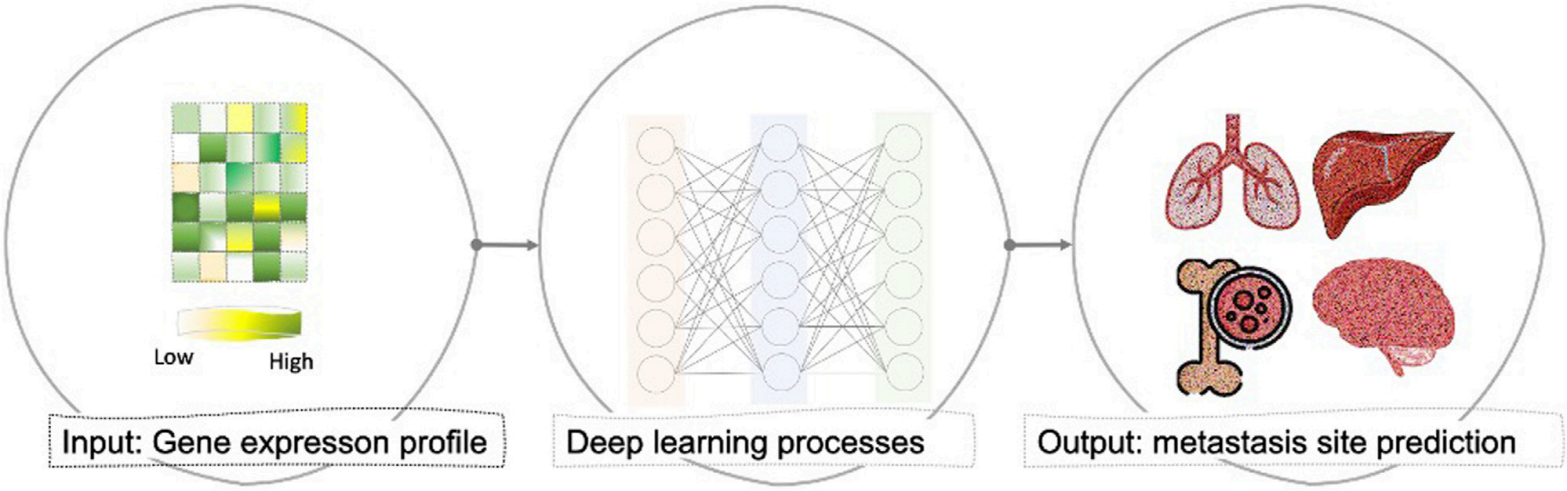
General overview of the proposed computational framework that takes the gene expression profile of any primary cancer sample and predicts whether patients’ samples are primary (localized) or had been metastasized to brain, bone, lung, or liver based on deep learning architecture.

In this work, we attempted to develop an AI method that could translate into a tool that supports clinical decision-making with regard to identifying metastasis and pinpointing the metastasis site (Figure 1). In this process, we also show the biological functions that the model uses to perform the prediction. That is, current work that interprets DL models identifies the genes that impact the prediction. Here, we propose interpreting the hidden neurons by linking the neurons to the enriched biological functions. In this work, we developed such a DL model. The DL framework takes as input raw gene expression data for a sample and predicts whether it is primary or metastasized to the brain, bone, lung, or liver. In the first phase, we used AutoEncoder (AE) to reduce the dimension of the expression data. Then, we applied DeepLIFT to compute an importance score (i.e., the impact of each input layer neuron on the latent layer neurons) used to rank the genes. Finally, to mine the genes that can distinguish the primary and tumor samples metastasized to different sites, we iteratively fed ten top-ranked genes (based upon the importance score) to the DNN model for training. In the second phase, we trained and evaluated a final multi-class DNN model to make the metastasis site predictions. Here, we also used the DeepLIFT approach to identify the essential neurons that lead to the prediction and the set of genes that activate these critical neurons. Then, we linked these critical genes to Gene Ontology (GO). We also provided analyses using Molecular Signatures Database (MSigDB) and the Disease Gene Network (DisGeNet) to support and increase the biology extracted from the essential neurons’ list of genes.

## 2 Method and materials

### 2.1 Gene expression datasets

We searched for gene expression datasets in Gene Expression Omnibus (GEO) (Edgar et al., 2002) using the following query: “metastas* AND (bone OR brain OR lung OR liver) AND Homo sapiens” filtered by “Expression profiling by array” in September 2021. We retrieved 837 entries which we sifted through and found microarray gene expression data for primary tumors (breast, colorectal, kidney, liver, lung, pancreatic, and prostate cancer samples), and tumors metastasized from these primary tumors to the bone, brain, lung, or liver. Table 1 provides the GEO accession numbers of the samples used in this study, along with the sample statistics. Similar to the approach used in (Chereda et al., 2019), we used the RMA probe-summary algorithm (Irizarry et al., 2003) to process each dataset, after which they were combined based on the HG-U133A array probe names, and quantile normalization was applied across all datasets. In cases where multiple probes were mapped to one gene, the probe with the highest average value was taken. Finally, we used the integrated datasets for each of the four sites as input for the DL models. However, before we fed the data to the DL model, we used the synthetic minority oversampling technique (SMOTE) to oversample the minority class using the imbalanced-learn python library (Chawla et al., 2002), as the number of samples is imbalanced between the primary and metastasized group.

**TABLE 1:** The gene expression datasets from GEO with the number of primary and metastasized samples for each site.

	Bone	Brain	Lung	Liver
Breast	220 Primary,	27 Primary,	47 Primary,	28 Primary,
	72 Metastasized [GSE 2034, GSE137842]	65 Metastasized [GSE12276, GSE125989, GSE46928, GSE18549]	18 Metastasized [GSE16554, GSE5327]	16 Metastasized [GSE18549]
Colorectal	0	10 Primary,	186 Primary,	219 Primary,
		23 Metastasized [GSE14108]	47 Metastasized [GSE18549, GSE41258]	86 Metastasized [GSE41258, GSE18549, GSE6605]
Kidney	0	0	10 Primary,	0
			10 Metastasized [GSE22541]	
Liver	0	0	31 Primary,	0
			31 Metastasized [GSE141016]	
Lung	14 Primary,	15 Primary,	0	0
	19 Metastasized [GSE10096]	23 Metastasized [GSE18549]		
Pancreas	0	0	0	15 Primary,
				14 Metastasized [GSE19279]
Prostate	16 Primary,	0	0	0
	17 Metastasized [GSE18549, GSE43332]			

### 2.2 Deep learning framework

**FIGURE 2:**
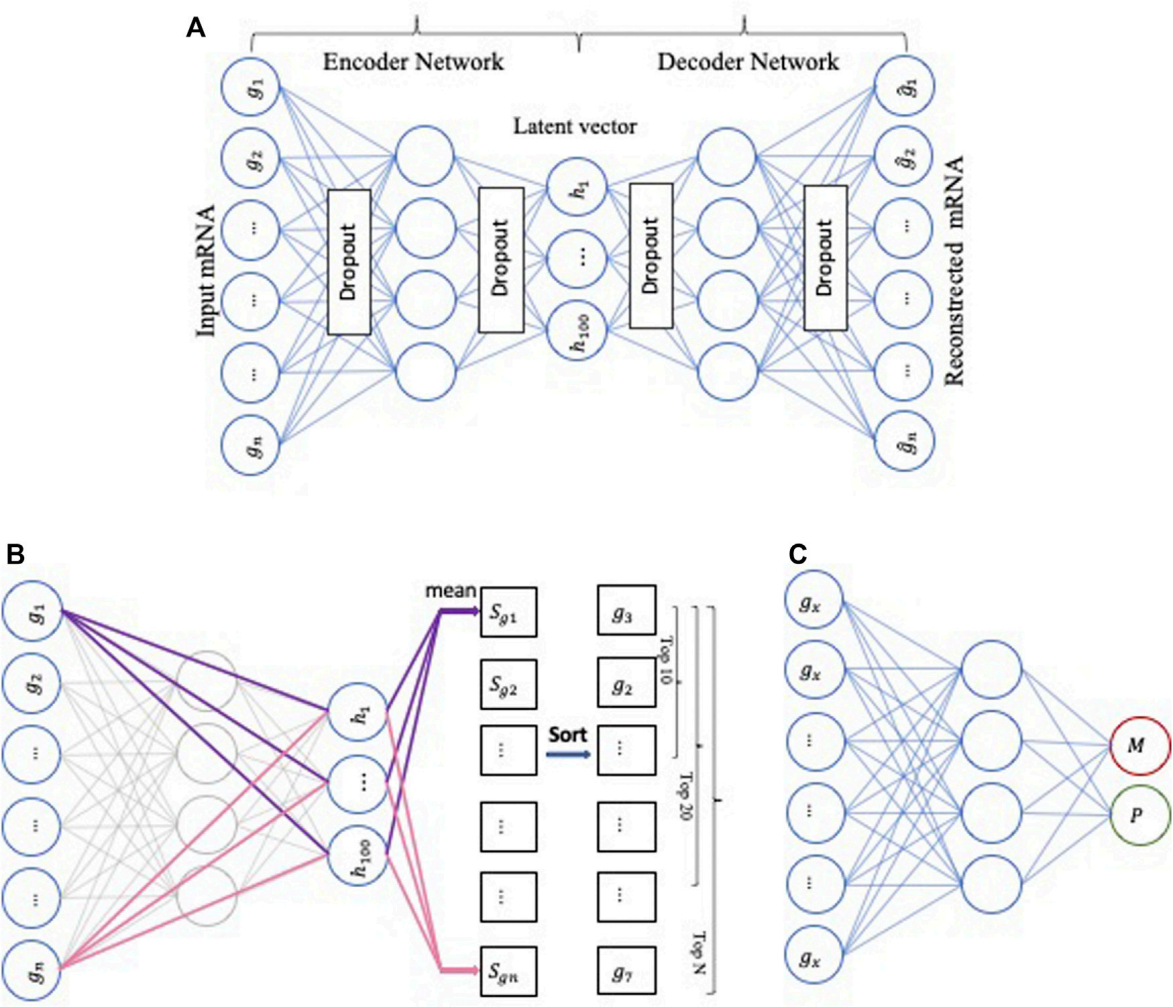
The workflow represents the first part of our model’s framework. **(A)** The architecture of AutoEncoder, **(B)** Applying DeepLIFT to compute the importance scores in the Encoder network, **(C)** Using DNN as a baseline method to perform the metastasis prediction.

The first part of our model’s framework comprises three key components, namely the AE (Hinton and Salakhutdinov, 2006), DeepLIFT (Shrikumar et al., 2017), and the deep neural network (DNN) (Svozil et al., 1997) (Figure 2).

First, the AE-based component is an unsupervised deep neural network with multiple stacked hidden layers composed of two parts, an encoder, and a decoder. The encoder maps the original (high-dimensional) data X to a reduced representation (100 dimensions) through the bottleneck layer. The purpose of the decoder is to reconstruct the original data $\hat{X}$ from the low-dimensional representation by minimizing the difference between X and $\hat{X}$ . In this manner, the AE extracts features that differ from the original features and functions as a feature extraction method. We used the Python Keras library (https://github.com/fchollet/keras) to implement an AE consisting of three fully connected hidden layers containing 500, 100, and 500 neurons. For each layer, we used “relu” as the activation function. Given m samples, each has a gene expression profile containing n genes; the input vector is reconstructed through a series of matrix transformations of multiple network layers. Training an AE involves finding parameters that minimize a specific loss function; we used mean absolute error (MAE) as the loss function. In addition, we added an L2 regularization penalty to control overfitting and used the early stopping technique. Finally, we trained the AE using the Adam (Kingma and Ba, 2014) optimization algorithm with 500 epochs and a 10% dropout.

Second, the DeepLIFT-based component is a feature scoring algorithm to calculate the contribution scores of each neuron. In our computing framework, we used DeepLIFT to calculate a contribution score for every gene of each input sample. The obtained contribution scores express the importance of the corresponding genes for the compression features of the low-dimensional representation (bottleneck) layer. Then, we ranked the genes based on their importance scores.

Third, the DNN-based component is a neural network with three hidden layers with 64, 32, and 8 neurons, respectively, and uses “relu” as the activation function. We used the Python Keras library to design the DNN model to predict if a sample is primary or metastasized. Finally, we iteratively added ten top-ranked genes (based on the importance scores) to train the DNN model.

**FIGURE 3:**
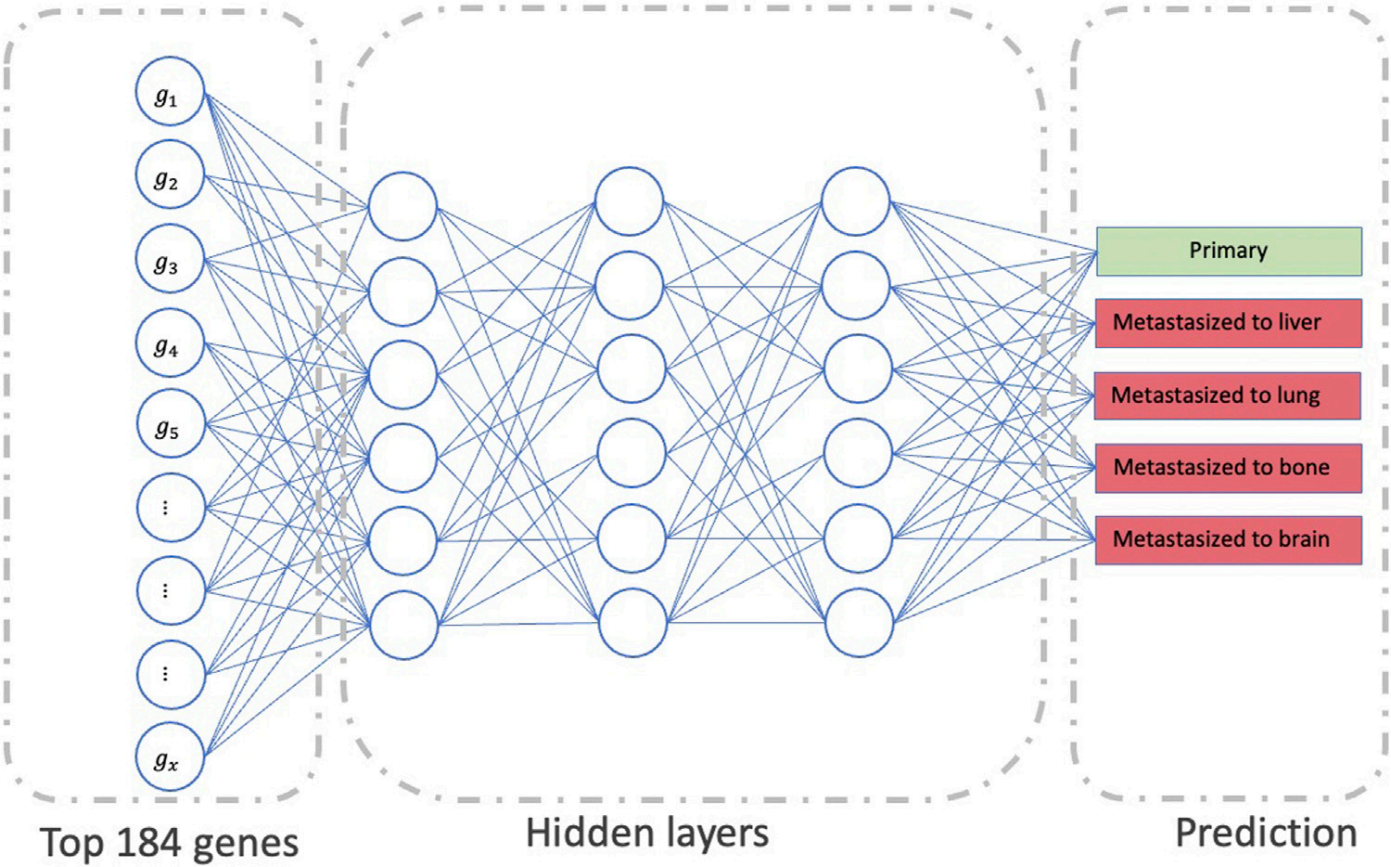
The workflow represents the second part of our model’s framework, which determines the significant neurons in the network to predict metastasis status.

The second part of our model uses the output from the first part, i.e., the most important genes for all sites, as an input to the final multi-class DNN model (Figure 3). This multi-class DNN consists of three hidden layers, each with 100 neurons, and uses a “relu” activation layer followed by an output layer with five output neurons (one for each class: primary, and metastasized to bone, brain, lung, or liver) that use the soft-max function to do the prediction. We then used the DeepLIFT to identify the most relevant neurons in each hidden layer for each of the five predictions (see 2.3 for details). Finally, the model was implemented in Python v.3.6 scripting language (https://www.python.org/), using the Keras deep learning and DeepLIFT frameworks (Figures 2B,C). Concerning time complexity, the time needed to train the model was 20.4 min for around 100 epochs for all samples using a workstation with Linux Ubuntu 18.04.5 LTS Intel Xeon Platinum 8,176, 64-bit OS and two GPUs: Quadro and Titan, with CUDA version 11.0.

### 2.3 Identifying the biological functions that the DL model uses to perform the prediction

We interpreted the prediction for each class by first computing the relevance scores through the DL network and identifying the most essential neurons that allow predicting the class. Then, we connected each important neuron with the list of the input genes affecting the neuron activation. In this manner, we associated biological functions with each layer based on its essential neurons.

The first step is to identify the neurons that most influence the predictions for each class (Bach et al., 2015; Hanczar et al., 2020). For this, we computed the relevance scores *R* of all neurons using the Deep-LIFT approach for each predicted class at each layer. Next, we used the mean of these relevance scores to obtain the average relevance of neuron *i* in layer *L*, representing this neuron’s influence on the DL network to predict the class. The relevance score for neuron *i* in layer *L* is defined as the sum of incoming scores from each neuron *j* in layer *L+1*.

Finally, we ranked the neurons according to their average relevance scores and chose the most essential ones. Similar to (Hanczar et al., 2020), assuming that the average relevance scores follow a Gaussian distribution, we used the two-side t-test (*p*-value at 0.05 ) to determine each class’s most essential neurons in each layer.

For a given important neuron in layer *L*, its activation is back propagated using the Deep-LIFT approach to compute the relevance score of each input gene. We then identified the most critical inputs that have an impact on the activation of the neuron. Similar to identifying the essential neurons, we used a two-sided t-test to select the essential input genes.

The second step is to connect each essential neuron to biological functions from GO, signature gene-set from MSigDB, and diseases from DisGeNET. Finally, we used an R interface to the Enrichr database EnricherR (Kuleshov et al., 2016) to identify the over-represented functions in the list of genes connected with each important neuron.

## 3 Results and discussion

### 3.1 Determining the gene set that provides optimal prediction performance

**FIGURE 4:**
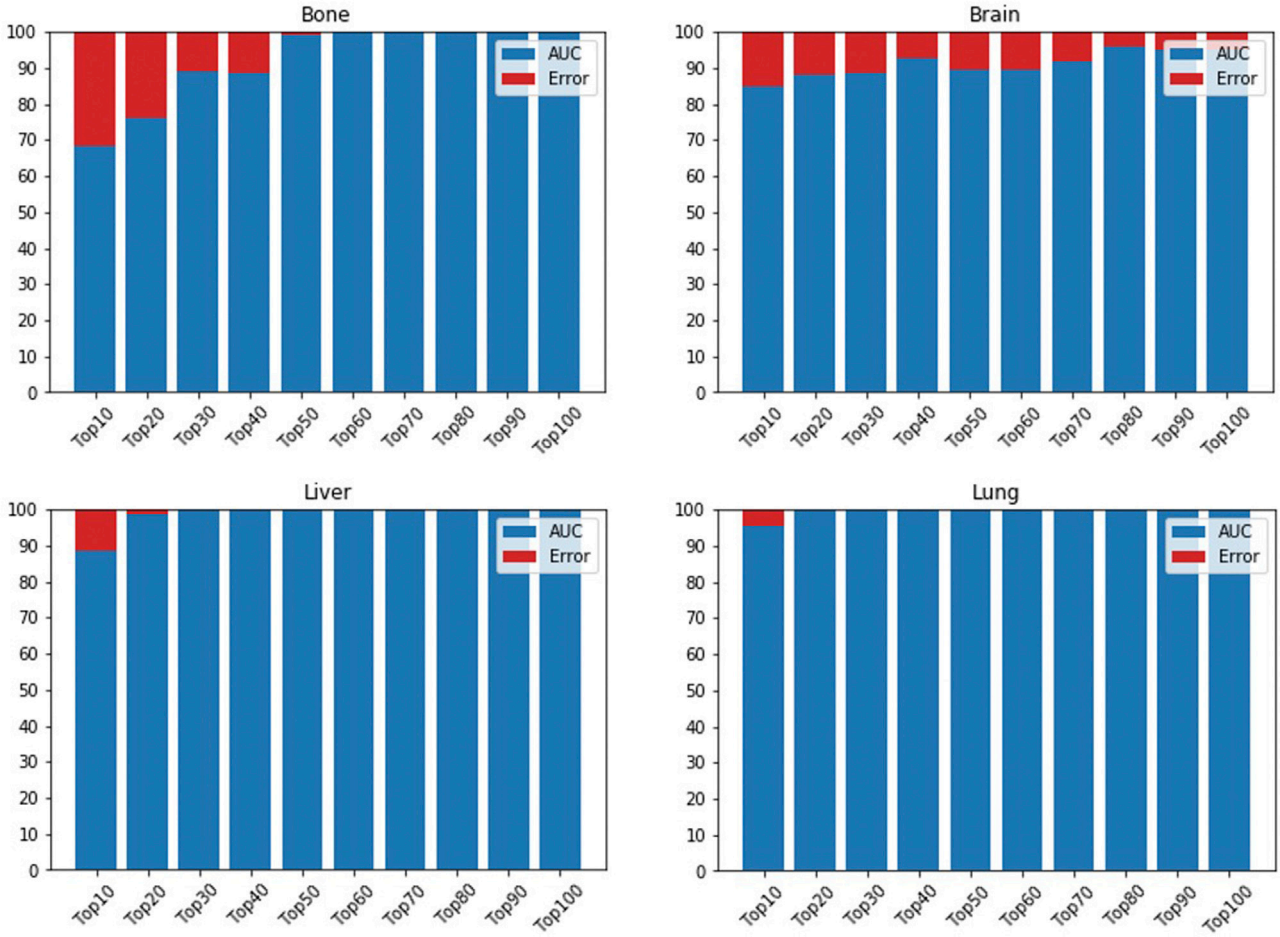
AUC is based on different numbers of featured genes using DNN for bone, brain, lung, and liver sites. AUC is indicated in blue, while error rate is shown in red.

In the first part of our model’s framework, we used GEO samples to train an AE and applied DeepLIFT to calculate importance scores for each gene for ranking. Then, ten top-ranked genes (based on the importance scores) were iteratively fed to the DNN model to identify the gene set that provides maximum performance when determining if a sample is primary or metastasized. The DNN reaches its maximum performance when including 60, 80, 20, and 30 top-ranked genes in metastasis to bone, brain, lung, and liver data, respectively (Figure 4). For the metastasis to bone, lung, and liver samples, the DNN achieved an AUC of 1.0. However, the DNN could only achieve an AUC of 0.9597 for the metastasis to brain samples. This might result from the brain samples having less than 50 primary samples, while the metastasis to bone, lung, and liver samples were analyzed using more than 200 primary samples. Adding weight to this suggestion is the number of metastasized samples used for each site being relatively the same (about 100 samples each).

### 3.2 Cross-site generalization analysis

**FIGURE 5:**
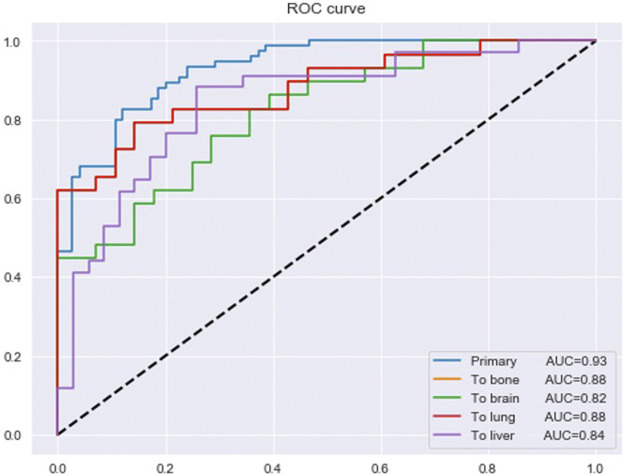
The prediction performance of the final multi-class DNN model.

After removing the duplicates, the 190 essential genes for all sites identified in the part of the model’s framework were reduced to 184 genes. The 184 genes were used as an input to the final multi-class DNN model. This model takes these 184 genes and predicts if the input samples are primary or metastasized to the bone, brain, lung, or liver site. Figure 5 provides the prediction performance for the final multi-class DNN model. The best prediction performance was achieved for the primary samples (AUC of 0.93), followed by the metastasis to bone and lung samples (AUC of 0.88). The metastasis to liver and brain samples achieved lower prediction performances with an AUC of 0.84 and 0.82, respectively. Here, we expected the prediction performance for metastasis to the brain to be the lowest, based on the maximum performance the DNN achieved in Figure 3. However, the final multi-class DNN model achieved a more than acceptable prediction performance in all categories.

#### 3.2.1 Testing the robustness of the final DNN model

**FIGURE 6:**
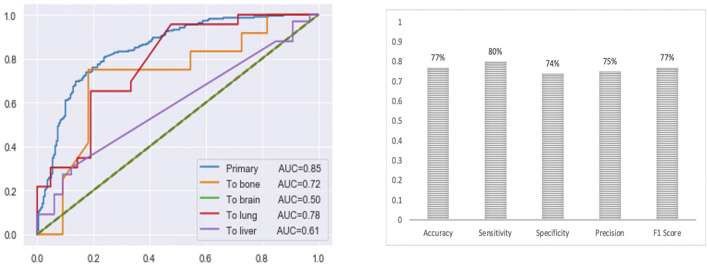
The prediction performance of the final multi-class DNN model using external testing data from the TCGA datasets. Note, for the brain there are only 2 samples in the test set).

**FIGURE 7:**
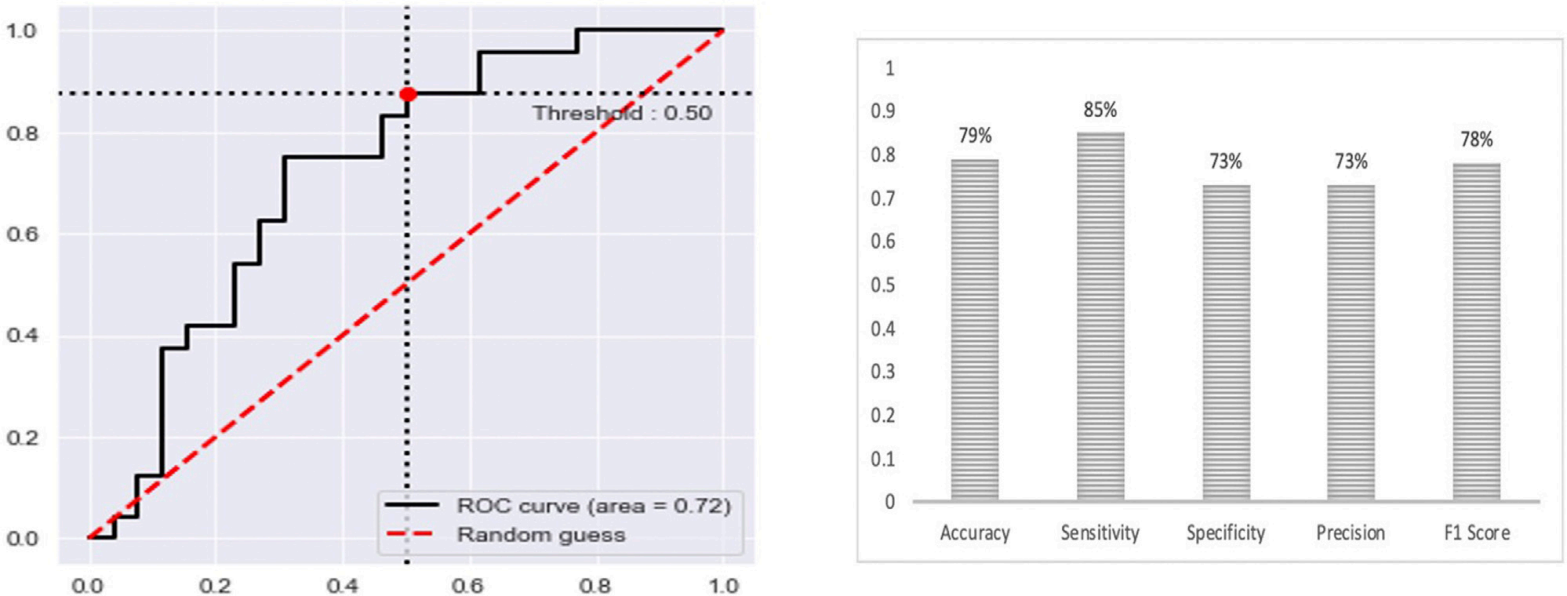
The prediction performance of the final multi-class DNN model using a specific population-based cohort.

The final multi-class DNN model achieved a good prediction performance; however, the prediction performance does not indicate the robustness of the final DNN model. Thus, we further evaluated the model’s performance using external testing data from the TCGA datasets (Figure 6) and using a population-based cohort (Figure 7). Also, by using external datasets as a validation technique to show how accurately our predictive model will perform in practice, we eliminate any concerns about over/under -fitting. First, the external set was extracted from the human cancer metastasis database (HCMDB) (Zheng et al., 2018), where we found 378 samples, 250 primary, and 21,2, 44, and 61 were metastasized to bone, brain, lung, and liver (see the complete list of TCGA IDs in Supplementary Table S1), respectively. In addition, we used the gene expression profiles of fresh breast cancer tissue of 45 (21 primary and 24 metastasized) Saudi-Arabian subjects deposited on GSE36295 to test the performance of our model on a population-based cohort (real data).

Figure 6 provides the prediction performance using the external set in terms of the area under the ROC curve and shows several other metrics, including accuracy, sensitivity, specificity, precision, and F1 score, ranging between 74%–80%. The prediction performance using the external data followed the same trend with the highest prediction performance achieved for the primary (AUC of 0.85) samples followed by the metastasis to lung (AUC of 0.78), bone (AUC of 0.72), liver (AUC of 0.61) and brain (AUC of 0.50) samples, respectively. This result shows that the multi-class model exhibits robustness concerning the three categories: the primary and metastasis to lung and bone samples. However, the prediction performance for the metastasis to the brain and liver samples dropped by 32% and 23 %, respectively. This suggests that we may have to re-establish the gene set that provides maximum performance using a larger cohort of samples (when the samples become available). Beyond that, here it should also be taken into consideration that for the brain we only had two samples in the test set.

Nonetheless, the prediction performance using samples from a population-based cohort shows that the multi-class DNN model achieved good prediction performance based on area under the ROC curve (AUC of 0.72), when distinguishing between the primary and metastatic samples (Figure 6), and shows several other metrics, including accuracy, sensitivity, specificity, precision, and F1 score, ranging between 73%–85%. This result gives an indication of the potential of our model to accurately predict metastasis sites.

#### 3.2.2 The biological functions associated with the genes used by the DL model to perform the prediction

**FIGURE 8:**
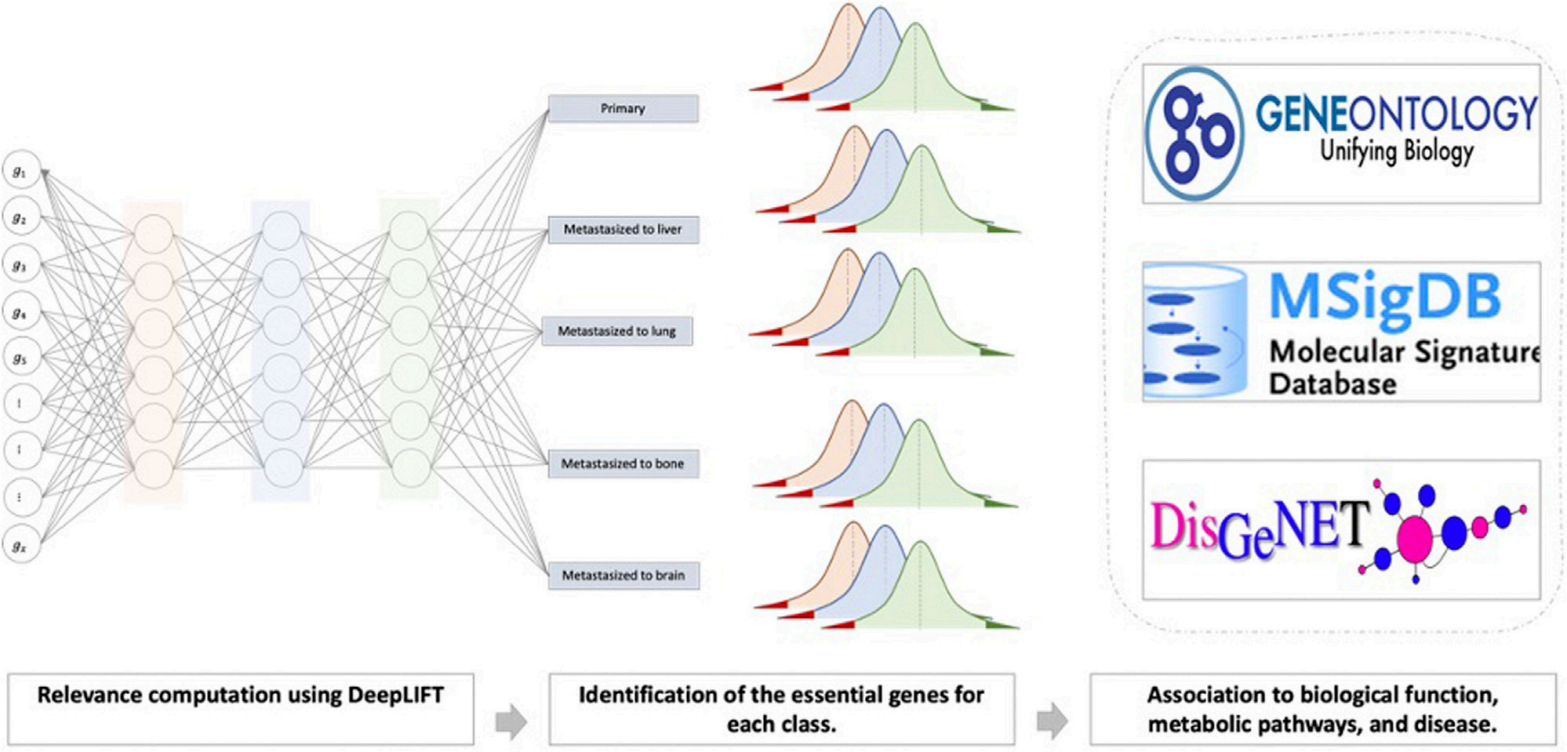
The biological interpretation of our deep neural network approach.

We further designed the model’s workflow to provide a second functionality beyond metastasis site prediction, i.e., to identify the biological functions that the DL model uses to perform the prediction (Figure 8).

We achieved this through the biological interpretation of the neural network predicting the metastasis. That is, for each class, the essential neurons of each layer are selected based on the mean of the relevance scores by using the method described in Section 2.3. We identified 89, 56, 41, 16, and 53 essential neurons in hidden layer one for primary, metastasized to bone, brain, lung, and liver, respectively. We also identified 36, 40, 99, 11, and 18 essential neurons in hidden layer two for primary, metastasized to bone, brain, lung, and liver, respectively. Finally, we identified 54, 48, 22, 84, and 35 essential neurons in hidden layer three for primary, metastasized to bone, brain, lung, and liver, respectively.

**FIGURE 9:**
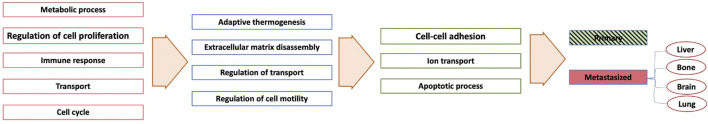
A simplified network showing each layer’s enriched pathways based only on the metastasis sites.

For each essential neuron’s list of genes, we determined GO biological functions based on a *p*-value < 0.05. Figure 9 provides the GO biological functions associated with the list of genes used to differentiate the primary samples from the metastasized ones. The critical neurons in each layer can be grouped depending on the functions enriched among the significant genes they contain. Overall, the enriched functions in layer one belonged to five main categories: “Metabolic process,” “Cellular process,” “Immune response,” “Transport”, and “Cell cycle.” The enriched functions from the essential neurons of layer two included “Adaptive thermogenesis,” “Extracellular matrix disassembly,” “Regulation of transport,” and “Regulation of cell motility.” The enriched functions from the essential neurons of layer three belonged to “Cell-cell adhesion,” “Ion transport,” “Apoptotic process.” The first layer exhibits more general function categories but more specific functions are appearing in subsequent neural network layers.

To support and increase the biological insights extracted from the essential neurons’ list of genes, we also performed MSigDB enrichment. In this analysis, we only considered MSigDB enrichments significant to at least three metastasis sites. Only three enriched categories were significant to all four sites, namely “Epithelial Mesenchymal Transition,” “Apoptosis,” and “IL-2/STAT5 Signaling” (Table 2). The apoptosis category is enriched based on the interpretation of the neural network (Figure 9) and the MSigDB enrichment. This is interesting as metastasis cells are subjected to various apoptotic stimuli and epithelial-mesenchymal transition (EMT) (which also features in the MSigDB enrichment) allows a polarized epithelial cell to undergo several biochemical changes to become a mesenchymal cell phenotype with enhanced resistance to apoptosis and increased migratory capacity and invasiveness and production of ECM components (Jason et al., 2003; Kalluri and Weinberg, 2009). Specifically, extracellular matrix disassembly (a GO biological function highlighted by the neural network) enzymes facilitates the remodeling of the extracellular matrix to create a microenvironment in the distant organ that promotes metastasis (Scheau et al., 2019; Winkler et al., 2020). Cell-cell adhesion, another GO biological function highlighted by the neural network, is also a key element of metastasis. For example, it has been shown that S100A8/A9 from tumor cells bind to RAGE on myeloid-derived suppressor cells (MDSCs) and promotes the migration and accumulation of MDSC, while periostin from MDSCs participates in pre-metastatic niche (PMN) formation through promoting extracellular matrix remodeling to facilitate the metastatic colonization of disseminated tumor cells (Cheng et al., 2008; Sinha et al., 2008; Wang et al., 2016). Overall, these biological functions suggest the gene lists used in the DL model to perform the prediction are to a large extent metastasis-specific and can be used to retrieve metastasis-specific biological functions beyond its metastasis site prediction capabilities (Sinha et al., 2008).

**TABLE 2:** MSigDB enrichment analysis.

	*p*-value			
MSigDB	Bone	Brain	Lung	Liver
Allograft rejection	2.60E-02	5.27E-03	Na	3.60E-03
Apoptosis	2.15E-02	2.00E-03	2.09E-02	2.15E-02
Coagulation	1.41E-02	na	1.82E-02	1.88E-02
DNA repair	1.53E-02	na	1.27E-02	2.02E-02
Epithelial mesenchymal transition	2.60E-02	1.98E-02	1.66E-02	2.60E-02
Glycolysis	2.60E-02	na	2.53E-02	3.11E-02
IL-2/STAT5 signaling	2.59E-02	2.02E-03	2.52E-02	2.59E-02
Interferon alpha response	na	1.39E-03	8.68E-04	1.39E-03
mTORC1 signaling	2.60E-02	na	1.66E-02	2.60E-02
Oxidative phosphorylation	3.60E-03	na	1.37E-03	3.28E-04
UV response up	2.12E-02	1.63E-02	2.06E-02	Na

a Enrichment associations that are significant to at least three sites

We also performed DisGenNET enrichment. In this analysis, we only considered DisGenNET disease enrichments significant to at least two metastasis sites. Only eight enriched disease categories were significantly associated with at least two metastasis sites, namely Autoimmune Diseases, Carcinoma breast stage IV, Cirrhosis, Dermatomyositis, Giant Cell Tumors, Leukemia, Metastatic malignant neoplasm to brain, and Rheumatoid Arthritis (Table 3). Four disease categories are associated with cancer, and noteworthy is the late-stage and metastasized cancer that is being picked up. Beyond this, Dermatomyositis (Luu et al., 2015), Rheumatoid Arthritis (Racanelli et al., 2008), and Autoimmune Diseases (Milkiewicz et al., 1999) are recognized paraneoplastic syndromes, which are symptoms that occur at sites distant from a tumor or its metastasis site (Pelosof and Gerber, 2010). In addition, several of our differentially expressed genes, including HLA-DMA, SOCS1, HLA-C, CTNNB1, KRAS, MET, and CD244, are associated with Liver Cirrhosis (Knouse et al., 2019), CD79A, HLA-DMA, SOCS1, HLA-B, HLA-C, IFI35, CD68, MET, PTHLH, CD244, and C2 with Rheumatoid Arthritis (Roy et al., 2011), HLA-B, HPRT1, and C2 with dermatomyositis (Bonnetblanc et al., 1990), and RB1, HLA-DMA, CXCR4, and CTGF with Cirrhosis (Shah and Casciola-Rosen, 2015). We also have several genes, including FTO, HLA-DMA, GAP43, SCN8A, HLA-C, CD68, and CDR2, associated with Multiple Sclerosis (Plantone et al., 2015), which suggest Multiple Sclerosis and Cirrhosis may possibly be a paraneoplastic syndrome that arises with metastasis.

**TABLE 3:** DisGeNET enrichment analysis.

	*p*-value			
DisGeNET	Bone	Brain	Lung	Liver
Autoimmune Diseases	5.12E-04	na	6.43E-05	Na
Carcinoma breast stage IV	2.68E-05	na	4.90E-04	Na
Cirrhosis	3.80E-04	na	0.00E+00	1.37E-02
Dermatomyositis	2.57E-05	2.57E-05	Na	Na
Giant Cell Tumors	5.69E-06	na	7.84E-08	Na
leukemia	7.13E-05	7.13E-05	Na	Na
Metastatic malignant neoplasm to brain	1.84E-04	na	3.43E-07	Na
Rheumatoid Arthritis	4.84E-05	na	2.71E-06	Na

a Disease associations that are significant to at least two sites

We further determined the overlapping genes between the primary and metastasis samples for the four sites. This analysis includes only the genes used by the DL to perform the classification. If we only considered genes common to at least three sites, we found the products of two genes, HIP1 and LARP4, with expression levels downregulated in the primary samples but upregulated in the metastasis samples. HIP1 was used by the DL to predict metastasis to the bone, brain, and lung, while LARP4 was used to predict metastasis to the brain, lung, and liver. This is interesting as HIP1 is one of the essential proteins involved in clathrin-mediated endocytosis (CME) (Chang et al., 2015), and crosstalk between CLCb/Dyn1-mediated adaptive CME and epidermal growth factor receptor (EGFR) signaling increases metastasis (Chen et al., 2017). Also, LARP4, a known RNA-binding protein (RBP) (Yang et al., 2011; Chothani et al., 2019) that repress or activate the translation of target genes, change the cell shape (which has been correlated with metastatic potential) and LARP4 depletion increases cell migration and invasion (Lyons et al., 2016; Seetharaman et al., 2016). Other proteins also upregulated and common to at least three sites (but do not appear in the primary samples gene list) include CC2D1A (Kumar et al., 2019), CD68 (Huang et al., 2018), EFCAB1 (Fagone et al., 2017), HLA-DMA (Li et al., 2020a), PRAME (Huang et al., 2016; Al-Khadairi et al., 2019), and ULBP2 (Paschen et al., 2009), all of which was linked to metastasis in previous studies. In fact, 87 % of the essential genes are associated with metastasis-related functions based on the current literature (Table 4).

**TABLE 4:** Literature linking the genes used by the DL to metastasis.

Gene	Link	Gene	Link	Gene	Link	Gene	Link
ACTC1	Ohtaki et al. (2017)	FTO	Ding et al. (2020)	NDUFC2-KCTD14	NA	RUBCN	Marsh and Debnath, (2020)
ADAM10	Xu et al. (2010)	GABARAP	Liu et al. (2021a)	NF1	Kitamura et al. (2010)	SCIN	NA
ANO1	Zhang et al. (2021a)	GAP43	Zhang et al. (2018a)	NOL3	Medina-Ramirez et al. (2011)	SCLY	Hartung et al. (2017)
ATP5PD	Song et al. (2016)	GAPDHS	Liu et al. (2017)	OCLN	Wang et al. (2018a)	SCN8A	(Hartung et al. (2017); Lopez-Charcas et al. (2018))
ATP5PO	McLaren and University of Western Australia, (2009)	GINS3	Li et al. (2021)	PACS2	Madreiter-Sokolowski et al. (2021)	SIGLEC1	Strömvall et al. (2017)
C2	NA	GNL3L	Kannathasan et al. (2020)	PCNX2	Yamaguchi et al. (2016)	SLC6A16	Nałęcz, (2020)
C5orf22	Schulten et al. (2017)	HIP1	Sun et al. (2021)	PFAS	Lv et al. (2020)	SNORD107	Xu et al. (2016)
C7orf25	NA	HIP2	Wu et al. (2020)	PIAS1	Wang et al. (2018b)	SNORD19B	Xu et al. (2016)
CC2D1A	Kumar et al. (2019)	HIP3	NA	PRAME	Huang et al. (2016)	SNORD42A	NA
CD244	Johnson et al. (2003)	HLA-B	(Cordon-Cardo et al. (1991); Jiang et al. (2014))	PRKACA	Honeyman et al. (2014)	SOCS1	David et al. (2014)
CD68	Huang et al. (2018)	HLA-C	Cordon-Cardo et al. (1991)	PRR14	Li et al. (2019a)	SSH3	Hu et al. (2019a)
CD79A	Luger et al. (2013)	HLA-DMA	Li et al. (2020a)	PTHLH	(Li et al. (2019a); Pitarresi et al. (2021))	SSX1	NA
CD82	Di Giacomo et al. (2017)	HPRT1	J Sedano et al. (2020)	RAB15	Iacobas et al. (2018)	ST20-MTHFS	NA
CD83	Giorello et al. (2021)	HPS4	Liu et al. (2018)	RAB26	Liu et al. (2021b)	SUOX	Yano et al. (2021)
CDR1	Harrison et al. (2020)	HSPA9	Yi et al. (2008)	RAD51B	Seguin et al. (2018)	TEP1	(Hwang et al. (2001); Yano et al. (2021))
CDR2	Balamurugan et al. (2009)	IFI35	Hu et al. (2021)	RB1	Ku et al. (2017)	TMSB4Y	Wong et al. (2015)
CHD1L	He et al. (2012)	IFITM2	Xu et al. (2017)	RHOB	Ju et al. (2020)	TPT1P8	NA
CHRNA1	Chang et al. (2013)	JAM3	(Xu et al. (2017); Zhou et al. (2019a))	RHOBTB2	Ling et al. (2010)	TREX1	Feng et al. (2016)
CTGF	Okusha et al. (2020)	KRAS	Boutin et al. (2017)	RPL13	Ebright et al. (2020)	TUBA3C	Zhou et al. (2019b)
CTNNB1	Wen et al. (2019)	KRT1	Han et al. (2021)	RPL21	Li et al. (2020b)	TUBGCP3	NA
CXCR4	Zhang et al. (2021b)	LARP4	Egiz et al. (2019)	RPL9	Baik et al. (2016)	UBA6	Cheng et al. (2021)
EFCAB1	Fagone et al. (2017)	LCP1	Ge et al. (2020)	RPP30	NA	UBD	Cheng et al. (2021)
ERCC3	Zhang et al. (2020)	LDHAL6B	(Ge et al., 2020; Liu et al., 2020)	RPS24	Wang et al. (2020)	ULBP2	Cheon et al. (2011)
ESR2	Song et al. (2018)	MET	Zhang et al. (2018b)	RPS6KA2	NA	USP6	Zeng et al. (2018)
FAM153A	NA	MME	Li et al. (2019b)	RPS8	Mao-De and Jing, (2007)	ZNF236	NA
FAXDC2	NA	MMP	Gonzalez-Avila et al. (2019)	RRP12	Hu et al. (2019b)	ZNF764	NA
FGF23	Ewendt et al. (2020)	NBEAL2	Rae et al. (2015)				

## 4 Concluding remarks

Metastasis remains the leading cause of cancer-related deaths worldwide, and our inability to identify the tumor cells colonizing distant sites means that the physician cannot treat the metastasized tumors. Here, we developed a DL model that can be fed raw gene expression data to predict whether a sample is primary or metastasized to the brain, bone, lung, or liver. The final multi-class DNN model achieved more than acceptable prediction performance in all categories. We achieved the best prediction performance for the primary samples (AUC of 0.93), followed by the metastasis to bone and lung samples (AUC of 0.88). On the other hand, the metastasis to liver and brain samples achieved lower prediction performance with an AUC of 0.84 and 0.82, respectively. We observed the same trend when evaluating the prediction performance using external data, i.e., the highest prediction performance for the primary (AUC of 0.85) samples followed by the metastasis to lung (AUC of 0.78), bone (AUC of 0.72), liver (AUC of 0.61) and brain (AUC of 0.50) samples, respectively. However, the prediction performance for the metastasis to the brain and liver samples dropped by 32% and 23 %, respectively.

Many factors may contribute to the result we obtained for the brain samples, as this data had the highest number of DEGs and required the highest amount of top-ranked genes to be included in the model, indicating biological complexity associated with the metastasis to the brain. Additionally, the brain samples had less than 50 primary samples. In contrast, we analyzed the metastasis to bone, lung, and liver samples using more than 200 primary samples (the number of metastasized samples used for each site was similar, about 100 samples each). Beyond that, the brain only had two samples in the test set for the external data that exhibited the massive drop in prediction performance. Having this lower number of brain samples may also be contributing to the much lower prediction performance achieved with it. Thus, in the future, we will re-establish the gene set that provides maximum performance using a larger cohort of samples (when the data become available). Nonetheless, we further evaluated the prediction performance using samples from a population-based cohort to show that the multi-class DNN model achieved good prediction performance (AUC of 0.72) when distinguishing between the primary and metastatic samples, which shows the potential of our model.

We further designed the model’s workflow to provide a second functionality beyond metastasis site prediction, i.e., to identify the biological functions that the DL model uses to perform the prediction. We achieved this by associating GO biological functions (*p*-value < 0.05) with the neuron’s list of genes that differentiate the primary samples from the metastasized ones in the DL model. The critical neurons in each layer are grouped depending on the functions enriched. Thus, the first layer exhibits more general function categories, but more specific functions appear in subsequent neural network layers. Finally, we compared the enrichments retrieved through the DL model neuron interpretations with the MSigDB enrichment analysis. We found only a few functional categories common to both analyses but several inter-related categories. For example, the literature shows “Epithelial Mesenchymal Transition’ involves ‘Ion transport,” and “Extracellular matrix disassembly,” and it is linked to “Cell-cell adhesion,” “regulation of cell motility” and “apoptosis process” (Jason et al., 2003; Cheng et al., 2008; Sinha et al., 2008; Kalluri and Weinberg, 2009; Wang et al., 2016; Scheau et al., 2019; Winkler et al., 2020). Overall, these biological functions suggest that the gene lists used in the DL model to perform the prediction are to a large extent metastasis-specific, which is further supported by literature showing 87% of the genes used by the DL have already been linked to metastasis. These results clearly suggest that our DL model can be used to retrieve metastasis-specific biological functions beyond its metastasis site prediction capabilities.

## 5 Availability

We also developed a web server that the scientific community can access. The web-based tool, MetastaSite https://www.cbrc.kaust.edu.sa/metastasite/, provides a means to implement the final multi-class DNN model developed in the current study. It allows the users to predict the metastasis site (primary, metastasized to bone, brain, lung, or liver). The user needs to provide the raw gene expression for every sample.

## Data Availability

The original contributions presented in the study are included in the article/Supplementary Material, further inquiries can be directed to the corresponding authors.
